# Comparison of Mechanisms of Endothelial Cell Protections Between High-Density Lipoprotein and Apolipoprotein A-I Mimetic Peptide

**DOI:** 10.3389/fphar.2019.00817

**Published:** 2019-07-19

**Authors:** Wenqi Xu, Mingming Qian, Caihua Huang, Pengfei Cui, Wei Li, Qian Du, Shenghui Yi, Xiaohe Shi, Yansong Guo, Jianlan Zheng, Donghui Liu, Donghai Lin

**Affiliations:** ^1^Key Laboratory for Chemical Biology of Fujian Province, MOE Key Laboratory of Spectrochemical Analysis & Instrumentation, College of Chemistry and Chemical Engineering, Xiamen University, Xiamen, China; ^2^Department of Cardiology, The Affiliated Cardiovascular Hospital of Xiamen University, Medical College of Xiamen University, Xiamen, China; ^3^Exercise and Health Laboratory, Xiamen University of Technology, Xiamen, China; ^4^Department of Cardiology, Fujian Provincial Hospital, Provincial Clinical Medicine College, Fujian Cardiovascular Institute, Fujian Provincial Key Laboratory of Cardiovascular Disease, Fujian Provincial Center for Geriatrics, Fujian Medical University, Fuzhou, China; ^5^Department of Ob/Gyn and Neonatal and Reproductive Medicine, The People’s Liberation Army 174th Hospital and The Affiliated Hospital of Xiamen University, Xiamen, China

**Keywords:** high-density lipoprotein, apoA-I mimetic peptide, oxidized low-density lipoprotein, endothelial cell, metabolomics

## Abstract

Apolipoprotein A-I (apoA-I) mimetic peptide, D-4F, exhibits anti-atherogenic effects similar to high-density lipoprotein (HDL). However, it remains elusive whether D-4F and HDL share similar molecular mechanisms underlying anti-atherogenic effects and endothelial cell protections. We here compared the metabolic changes in endothelial cells induced by D-4F and HDL against oxidized low-density lipoprotein (ox-LDL), which may be of benefit to understanding the protective mechanisms of HDL and D-4F. Functional assays, including wound healing, transwell migration, and tube formation, were used to evaluate the pro-angiogenic effects of HDL and D-4F. NMR-based metabolomic analysis was employed to explore the protective mechanisms underlying HDL and D-4F. Partial least-squares discriminant analysis (PLS-DA) was performed to assess metabolic profiles, and orthogonal PLS-DA (OPLS-DA) was carried out to identify characteristic metabolites. Moreover, significantly altered metabolic pathways were also analyzed. We found that ox-LDL impaired the migration and tube formation of endothelial cells. Metabolomic analysis showed that ox-LDL triggered oxidative stress, impaired glycolysis, and enhanced glycerophospholipid metabolism. Both HDL and D-4F improved the migration and angiogenesis of endothelial cells, alleviated oxidative stress, and ameliorated disordered glycolysis impaired by ox-LDL. Strikingly, HDL partially attenuated the disturbed glycerophospholipid metabolism, whereas D-4F did not show this effect. In summary, although D-4F shared the similar protective effects with HDL on the migration and angiogenesis of endothelial cells, it could not deduce the molecular mechanisms of HDL completely. Nevertheless, D-4F possesses the potentiality to be exploited as clinically applicable agent for endothelial cell protection and cardiovascular disease treatment.

## Introduction

High-density lipoprotein (HDL) is a complex composed of several bioactive proteins and lipids (Birner-Gruenberger et al., 2014), which makes it a potent therapeutic target for cardiovascular disease (CVD) owing to its pivotal anti-atherogenic functions (Rye and Barter, 2014; van Capelleveen et al., 2014). In addition to reverse cholesterol transport (RCT), HDL also exhibits several anti-inflammatory, anti-oxidative, and anti-apoptotic effects on endothelial cells (Rosenson et al., 2013; Kratzer et al., 2014). Moreover, HDL enhances the productions of nitrite oxide (NO) and prostaglandin I2 (PGI2), stimulates proliferation and migration of endothelial cells, and inhibits inflammation and apoptosis in endothelial cells (Riwanto and Landmesser, 2013). As an enzyme located almost exclusively in HDL, Paraoxonase 1 (PON1) is primarily responsible for the antioxidative functions of HDL (Soran et al., 2015). Besides, HDL in plasma is a major carrier of sphingosine-1-phosphate (S1P), which exhibits multiple biological activities (Levkau, 2015). More significantly, the major apolipoprotein of HDL, apolipoprotein A-I (apoA-I), exerts important protective effects on endothelial cells, making it to be an attractive agent for CVD treatment (Patel et al., 2010; Reimers et al., 2011). However, the clinical use of apoA-I is limited due to its large molecular composition, expensive manufacture, and intravenous administration.

ApoA-I mimetic peptides are a series of synthesized peptides, which share similar biological functions with native apoA-I (Navab et al., 2005). Among these mimetic peptides, 4F, containing 4 phenylalanine (F) residues, is extensively studied because it was shown to be the most effective in a chemotaxis assay system (Navab et al., 2005; White et al., 2014). D-4F is synthesized from all D-amino acids which cannot be degraded by digestive enzyme but be absorbed into plasma orally (Dunbar et al., 2017), and this property makes D-4F to be more potential for clinical application (Navab et al., 2002). Accumulating evidences indicated that D-4F protects endothelial cells against ox-LDL-induced injury by antagonizing the down-regulation of pigment epithelium-derived factor (PEDF) (Ding et al., 2014). We also found that D-4F alleviates ox-LDL-induced oxidative stress and promotes endothelial repair through the eNOS/HO-1 pathway (Liu et al., 2017a; Liu et al., 2017b). Furthermore, D-4F accelerates vasodilatation and restrains atherosclerosis by both regulating phospholipid metabolites and decreasing plasma long-chain lysophosphatidylcholine (LysoPC) in LDL-R null mice (Ou et al., 2012). Additionally, D-4F decreases the myocardial infarction area in hyperglycemia mice through promoting the release of NO and decreasing the generation of reactive oxygen species (ROS) in endothelial cells (Baotic et al., 2013).

Given the distinctly different compositions between HDL and D-4F, it is expected that different molecular mechanisms underlie the protective effects of HDL and D-4F. However, these molecular mechanisms still remain to be addressed in details. Recent findings unraveled that endothelial cell biology is driven by a metabolic switch, and metabolic alterations in endothelial cells could mediate angiogenesis and vasculature formation (Eelen et al., 2015; McGarrity et al., 2016). Additionally, as the final downstream products of transcription and translation, metabolites serve as the potential indicators of enzyme activities in pathological and pathophysiological conditions, and metabolomic assay might reveal the overall abnormal metabolic changes in CVD (Kordalewska and Markuszewski, 2015; Würtz et al., 2015). Thus, comprehensive metabolomic analysis might be a useful tool that would provide an in-depth insight into the different molecular mechanisms of HDL and D-4F underlying the predictive effects on endothelial cells.

Herein, we conducted a nuclear magnetic resonance (NMR)-based metabolomic analysis to investigate ox-LDL-induced metabolic disorders, and address different metabolomic changes of endothelial cells induced by HDL and D-4F, as well as reveal the distinct protective mechanisms underlying the protective effects of HDL and D-4F. Our results showed that although D-4F shared the similar protective effects with HDL on angiogenesis of endothelial cells, it could not fully encompass the molecular mechanisms of HDL related to endothelial cell metabolism. Although D-4F did not show the capacity of regulating glycerophospholipid metabolism, it shared several molecular mechanisms with HDL for regulating the metabolism of endothelial cells. Our work provides the mechanistic basis for exploring D-4F to be a clinically applicable agent for CVD treatment.

## Materials and Methods

### Cell Culture and Materials

Human umbilical vein endothelial cells (HUVECs) were isolated by collagenase-based digestion of umbilical veins from healthy donors (Liu et al., 2017a; Liu et al., 2017b). HUVECs were cultured in gelatin-coated polystyrene dishes with endothelial cell medium (ECM) supplemented with 5% fetal bovine serum (FBS) and endothelial cell growth supplement (ECGS) in an incubator with 5% CO_2_ at 37°C (Liu et al., 2011). The cells were used for experiments at passages 3–5. Before the treatments, HUVECs underwent serum starvation in 0.5% FBS-ECM for 6 h unless otherwise indicated.

D-4F (Ac-D-W-F-K-A-F-Y-D-K-V-A-E-K-F-K-E-A-F-NH2, purity 95%) was synthesized by China Peptides Co (Shanghai, China). Crystal violet was purchased from Sigma-Aldrich Co. (St. Louis, MO, USA). ECM (No. 1001) was purchased from ScienCell Research Laboratories (Carlsbad, CA, USA). Trypsin and FBS were procured from Gibco Co. (Carlsbad, CA, USA). Transwell chambers with 8-μm pore polycarbonate membrane were obtained from Millipore Co. (Billerica, MA, USA). Growth Factor Reduced Matrigel^™^ Matrix (No. 356230) was purchased from BD Medical Technology (Lake Franklin, NJ, USA). All other chemicals and reagents were obtained from commercial sources and were of analytical grade.

### Isolation of Lipoproteins

After overnight fasting, fresh blood from healthy volunteers was drawn into EDTA-Na_2_ (1 mg/ml) tubes and placed on ice. Plasma was separated by centrifugation at 2,500 rpm at 4°C for 15 min. LDL (1.019–1.063 g/ml) and HDL (1.063–1.210 g/ml) were isolated by ultracentrifugation as described previously (Liu et al., 2011). Briefly, the plasma density was adjusted to 1.3 g/ml with KBr, and normal saline (1.006 g/ml) was layered over the adjusted plasma to form a discontinuous NaCl/KBr density gradient. Subsequently, the tubes loaded with sample and gradient were subjected to ultracentrifugation in a P40ST rotor of a HITACHI ultracentrifuge (model: CP70MX, Japan) at 350,000×*g* for 4 h at 4°C. The LDL and HDL layers were collected separately. The particles of LDL and HDL were identified by agarose gel electrophoresis. Also, the purities of LDL and HDL were evaluated through gradient sodium dodecyl sulphate-polyacrylamide gel electrophoresis (SDS-PAGE) (Liu et al., 2011). Both LDL and HDL were analyzed by automatic biochemistry analyzer (HITACHI, Japan). ApoA-I was quantified by nephelometry (Dimension XPand, Dade Behring, Germany). LDL and HDL were dialyzed against phosphate-buffer saline (PBS), sterilized through a 0.22-mm filter, and stored in sealed tubes at 4°C in the dark for subsequent use.

### Oxidation of LDL

*In vitro* ox-LDL was prepared *via* exposure of LDL to 5 μM CuSO_4_ for 24 h at 37°C (Liu et al., 2017a; Liu et al., 2017b). The oxidation was terminated by adding EDTA-Na_2_ to a final concentration of 1 mg/ml. Then, ox-LDL was dialyzed against PBS for 48 h to remove EDTA-Na2 and sterilized again. The oxidation of LDL was tested by measuring thiobarbituric acid-reactive substances (TBARSs) (Liu et al., 2017a; Liu et al., 2017b).

### Transwell Migration Assay

Cell migration was conducted using a 24-well Transwell plate containing polycarbonate 8-μm pore membrane filters (Liu et al., 2017a; Liu et al., 2017b). HUVECs mixed with various doses of either HDL or D-4F were seeded in the upper wells (1 × 10^6^ cells/200 μl of ECM containing 0.5% FBS), whereas the lower wells were filled with 800 μl of ECM containing 5% FBS. After 8 h, ox-LDL (100 μg/ml) was added to each chamber, and HUVECs were incubated for another 8 h at 37°C. The migrated cells were fixed with 4% paraformaldehyde and stained with crystal violet. The number of cells migrated to the lower side of the filter was counted in six random fields (100×) per well using a light microscopy (Nikon, Tokyo, Japan).

### Scratch-Wound Assay

HUVECs were grown to 80% confluence in six-well plates, and then the monolayer of cells was scratched gently. Cells were rinsed twice with PBS to remove the cellular debris, and the linear wound was recorded. HUVECs were incubated with either HDL or D-4F for 12 h in ECM with 0.5% FBS, and subsequently treated with ox-LDL (100 μg/ml) for 8 h. The number of cells migrated into the wound space was manually counted in six random fields (100×) per well.

### Tube Formation

Ninety-six-well plates were coated with growth factor-reduced Matrigel Matrix (50 μl/well) and incubated at 37°C for 30 min to allow gelation to occur (Prosser et al., 2014). HUVECs were added to the top of the gel at a density of 25,000 cells/well in either the presence or absence of HDL or D-4F. Cells were grown at 37°C with 5% CO_2_ for 8 h in ECM with 1% FBS, and subsequently treated with or without ox-LDL (100 μg/ml) for 4 h. Tube formation was recorded with a light microscopy (100×) and analyzed using the ImageJ software.

### Intracellular Metabolite Extraction

In the metabolomic experiments, all the four groups of HUVECs (control, ox-LDL, HDL + ox-LDL and D-4F + ox-LDL) firstly underwent serum starvation and subsequently treated with either HDL (100 μg/ml) or D-4F (20 μg/ml) for 8 h in 0.5% FBS-ECM. Thereafter, these cells were continually treated with ox-LDL (100 μg/ml) for another 12 h. As the molar concentration of the bovine HDL in 0.5% FBS-ECM was much lower than that of D-4F in the medium, the influence of the bovine HDL on endothelial cells could be ignored.

Approximately 5 × 10^6^ cells in each group were harvested and quenched (Teng et al., 2009). After being washed thrice with ice-cold PBS (pH 7.4), the cells were immediately quenched with 3 ml of pre-cooled HPLC-grade methanol, and gently scratched using a cell scraper. The methanol solution containing cells was pipetted for extraction. Intracellular metabolites were extracted using a dual-phase extraction procedure (Viant, 2007). A mixture of methanol, chloroform, and ultrapure water in the volume ratio of 4:4:2.85 was applied to generate a two-phase extract. Only the aqueous phase was used for NMR-based metabolomic analysis.

### Sample Preparation and NMR Measurement

Before NMR analysis, solvents were completely removed using a Nitrogen Blowing Concentrator. The lyophilized intracellular metabolite extracts were resuspended in 500 μl of D_2_O and 50 μl of PBS (1.5 M K_2_HPO_4_/NaH_2_PO_4_ containing 0.1% TSP and 0.2% NaN_3_, pH 7.4), vortexed and centrifuged at 12,000×*g* for 15 min at 4°C to remove precipitates. Herein, D_2_O was used for field-frequency lock, and TSP was used to provide the chemical shift reference (δ 0.00). The supernatants (500 μl for each sample) were transferred to 5-mm NMR tubes (Beckonert et al., 2007). All ^1^H NMR spectra were recorded on a Bruker Avance III 850 MHz spectrometer (Bruker Bio Spin, Germany) at 25°C using the pulse sequence NOESYPR1D [(RD)-90°-t_1_-90°-τm-90°-acq] with water suppression. The spectral width was 20 ppm with an acquisition time per scan of 1.88 s, using a 4-s relaxation recovery delay between acquisitions. A total of 128 transients were collected into 64 K data points for each spectrum.

### NMR Data Preprocessing

NMR spectra were processed (phase correction and baseline correction) by using the MestReNova software (Version 6.1, Mestrelab Research S.L., ESpain). The spectral region δ 9.5 to 0.5 was segmented into chemical shift regions of equal width of 0.002 ppm. Integrals of the spectral region δ 5.5 to 4.5 were set to zero to eliminate the distorted baseline from imperfect water saturation. The icoshift algorithm was executed to remove misalignment of NMR signals in MatLab (Version 2011b; Math Works, USA). The integrals were normalized to the sum of the spectral intensity to compensate for the variations in sample concentration. Metabolites were identified by combining literatures (Tripathi et al., 2012; Shao et al., 2014), Chenomx NMR Suite (Version 7.7, Chenomx Inc., Edmonton, Canada), and Human Metabolome Data Base (HMDB, http://www.hmdb.ca/).

### Statistical Analysis for NMR and Functional Experiments

For multivariate analysis of the NMR data, the normalized spectral data were imported into the SIMCA-P software (Version 12.0; Umetrics AB, Umeå, Sweden) and scaled by Pareto scaling to enhance the importance of low-level metabolites without significant amplification of noise. The unsupervised PCA was performed to display general separation of metabolic profiles and identify the outliers (data not shown). Moreover, the supervised partial least-squares discriminant analysis (PLS-DA) and orthogonal PLS-DA (OPLS-DA) were conducted to improve the group separation. To avoid data overfitting, permutation tests with 200 iterations were carried out to validate the PLS-DA model. This validation was usually conducted by comparing the goodness of fit (R^2^ and Q^2^) of the original model with that of several models based on data where the order of the Y-observations was randomly permuted, while the X-matrix was kept intact. R^2^ represented the explanation of the model (R^2^X and R^2^Y denote the fraction of the variance of X matrix and Y matrix), while Q^2^ denoted the predictive accuracy of the model. The values of R^2^Y and Q^2^ close to 1.0 were indicative of a valid model with a high robustness. The variable importance in the projection (VIP) scores computed from the OPLS-DA model was taken as one of the criterions for identifying characteristic metabolites. Variables with VIP > 1.0 were considered relevant for group discrimination (Gu et al., 2016).

To relatively quantify the metabolite levels, we calculated the relative intensities of these identified metabolites from ^1^H NMR spectra, which were expressed as mean ± SEM. We made comparisons of the metabolite levels among the groups by conducting one-way ANOVA followed by *post hoc* test based on least significant difference (LSD) using the SPSS software (version 18.0, Chicago, IL, USA). The variations with *p* < 0.05 were considered as statistically significant. Characteristic metabolites were confirmed on the basis of *p* < 0.05 and VIP > 1.0.

The experiments of cell migration and tube formation were repeated at least three to four times. Differences were compared with either two-tailed Student’s *t* test or one-way ANOVA by using GraphPad Prism (Version 6.0, La Jolla, CA, USA). Data are expressed as mean ± SEM (*p < 0.05, **p < 0.01, ***p < 0.001).

### Metabolic Pathway Analysis

We identified significantly altered metabolic pathways based on the characteristic metabolites by combining two online software systems: 1) MetaboAnalyst (Version 3.0, http://www.metaboanalyst.ca/), a comprehensive web server for metabolomics data analysis, visualization and interpretation; and 2) Kyoto Encyclopedia of Genes and Genomes (KEGG, http://www.genome.jp/kegg/), a database resource that integrates genomics, chemical, and systemic functional information. The module of pathway analysis was offered by MetaboAnalyst, which integrates metabolite sets enrichment analysis and pathway topology analysis, using two criterions to identify the significant metabolic pathways, including probability mass function (p) and pathway impact value (PIV) as the cumulative percentage from the matched metabolite nodes. On the other hand, as a comprehensive knowledge repository, KEGG was extensively utilized as one of the main data resources to reconstruct metabolic networks (Kanehisa et al., 2008; Gu et al., 2016). The metabolic network can be viewed both as a network of proteins (metabolic enzymes) and as a network of chemical compounds (metabolites).

## Results

### Pro-Migratory Effects of HDL and D-4F on Endothelial Cells

**Figure 1:**
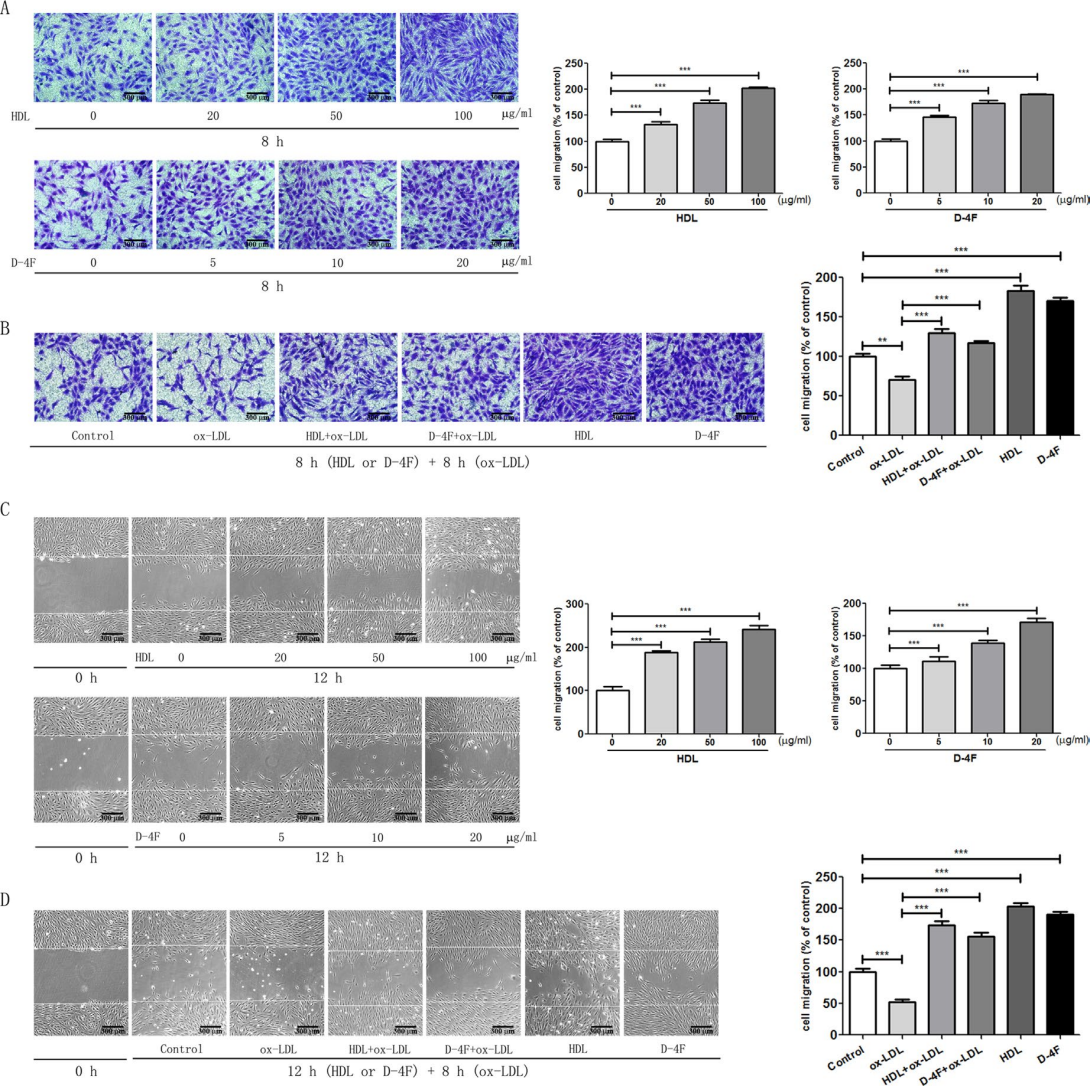
HDL and D-4F promoted the migration of endothelial cells impaired by ox-LDL. Endothelial cells were incubated with various doses of either HDL or D-4F **(A)** for 8 h for transwell assay, or **(C)** for 12 h for wound healing assay. Alternatively, endothelial cells were pre-incubated with either HDL (100 µg/ml) or D-4F (20 µg/ml) for 8 h **(B)** or for 12 h **(D)**, and further treated with ox-LDL (100 µg/ml) for 8 h for transwell assay or for wound healing assay. Migrated cells were photographed and counted in six random high-power fields (100×). The bar length was 300 µm. *p < 0.05; **p < 0.01; ***p < 0.001.

The results from the transwell chamber experiments showed that both HDL and D-4F induced HUVEC migration in a dose-dependent manner (**Figure 1A**; **Supplementary Table 1**). Moreover, the results from the transwell chamber experiments showed that both HDL and D-4F improved the migration of HUVECs impaired by ox-LDL (**Figure 1B**; **Supplementary Table 1**). Similarly, the results from the scratch-wound healing experiments also showed that both HDL and D-4F induced HUVEC migration in a dose-dependent manner (**Figure 1C**; **Supplementary Table 1**). Furthermore, the results from the scratch-wound healing experiments showed that both HDL and D-4F improved the migration of HUVECs impaired by ox-LDL (**Figure 1D**; **Supplementary Table 1**). These results illustrated that D-4F possessed the similar pro-migratory effects to HDL on endothelial cells either alone or even challenged by ox-LDL.

### Tube Formation of Endothelial Cells Promoted by HDL and D-4F

**Figure 2:**
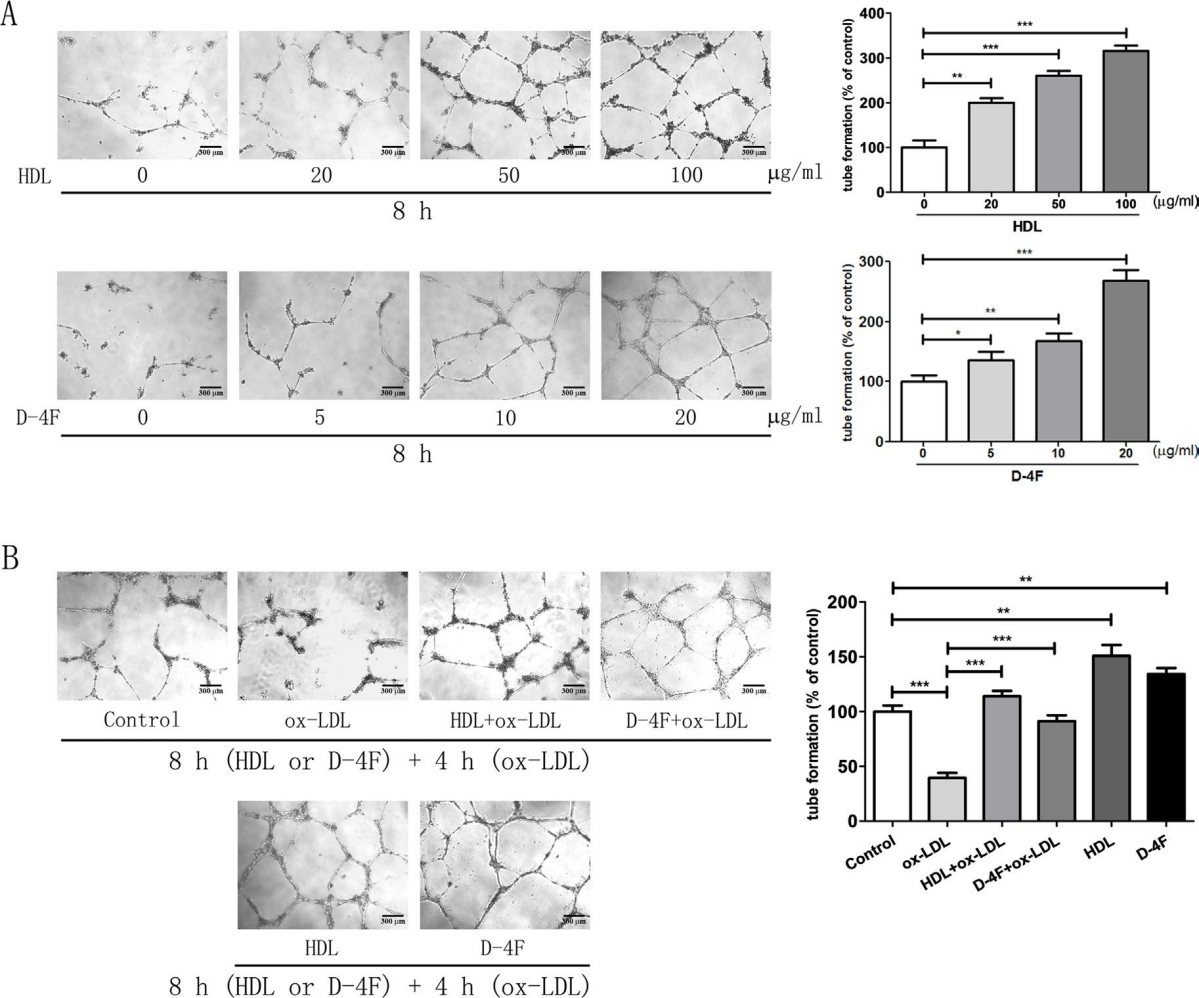
HDL and D-4F improved the tube formation of endothelial cells challenged by ox-LDL. **(A)** Endothelial cells were incubated with various doses of either HDL or D-4F for 8 h. **(B)** Alternatively, endothelial cells were pre-incubated with either HDL (100 µg/ml) or D-4F (20 µg/ml) for 8 h, and further treated with ox-LDL (100 µg/ml) for 4 h. Tube formation was recorded in Matrigel with a light microscopy (100×). The bar length was 300 µm. *p< 0.05; **p< 0.01; ***p< 0.001.

Consistently with those from the endothelial cell migration experiments, the results from the experiments of tube formation of endothelial cells displayed that both HDL and D-4F promoted HUVEC tube formation in a dose-dependent manner (**Figure 2A**; **Supplementary Table 2**). Furthermore, the results from the experiments of tube formation of endothelial cells also displayed that both HDL and D-4F alleviated the inhibitory effects of ox-LDL on HUVEC tube formation (**Figure 2B**; **Supplementary Table 2**). These results exhibited that D-4F possessed the similar pro-angiogenic effects to HDL *in vitro*.

### NMR Spectra and Pattern Recognition Analysis

**Figure 3:**
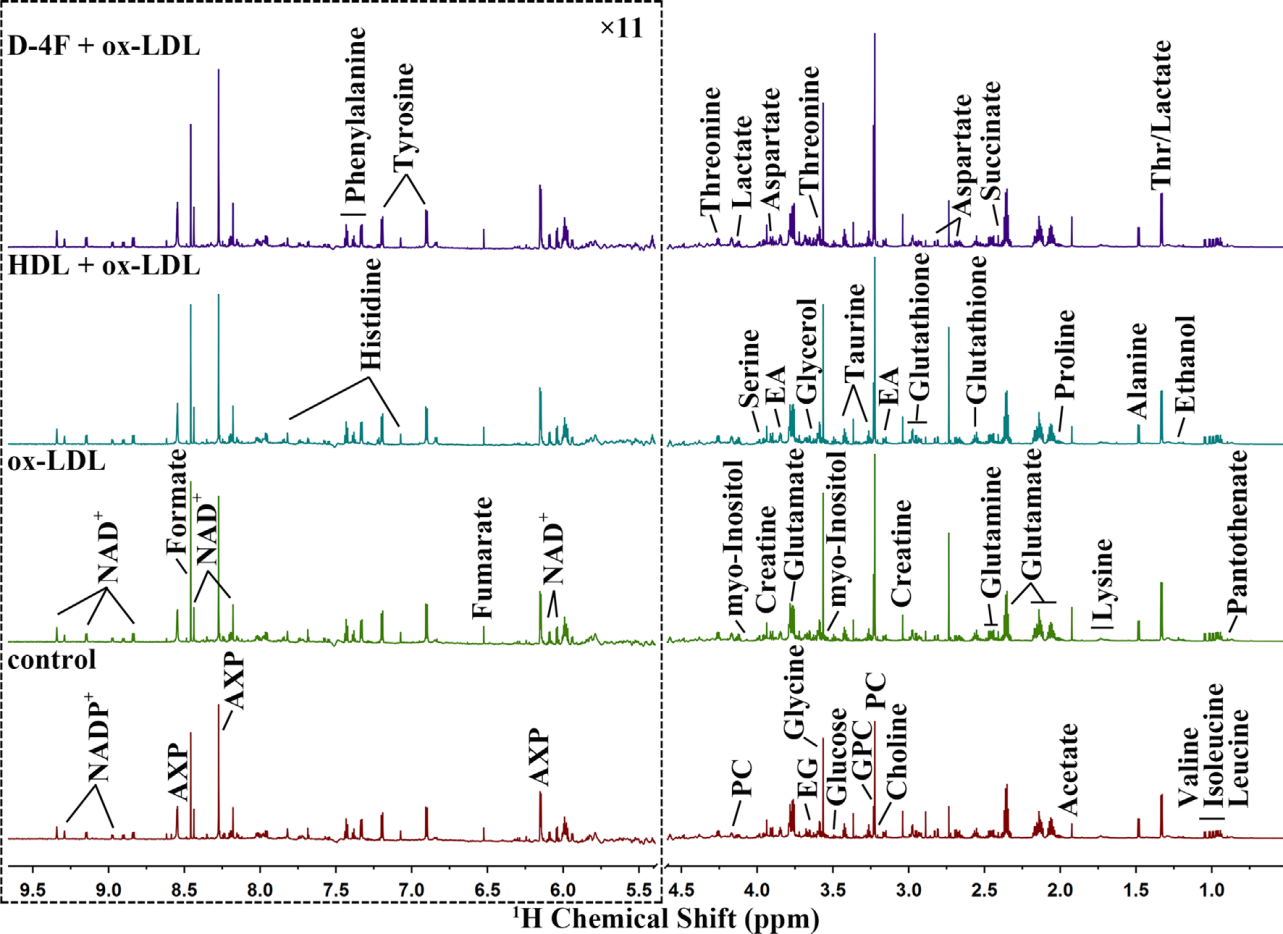
Average 850 MHz 1D ^1^H NMR spectra recorded on aqueous extracts derived from endothelial cells. The vertical scales were kept constant in all the ^1^H spectra. The water region (4.5–5.5 ppm) was removed. The region of 5.5–9.5 ppm (in the dashed box) was magnified 11 times compared with the corresponding region of 0.5 to 4.5 ppm for the purpose of clarity.

Averaged ^1^H NMR spectra obtained from aqueous extracts of HUVECs are shown in **Figure 3**, which display similar spectral profiles with a number of peak intensity discrepancies among the four groups (D-4F + ox-LDL, HDL + ox-LDL, ox-LDL, and control). Resonance assignments of metabolites were performed. Given that NMR-based metabolomic analysis is extensively used to detect small molecules with relative molecular weight ≤ 1 kDa, the aqueous solution contained mainly aqueous metabolites extracted from cells, including lipid metabolism-related metabolites, carbohydrates, amino acids, and other small metabolites. In addition, the aqueous solution did not contain the residual intracellular HDL or D-4F, which was denatured by methanol in the process of cell disruption.

Totally, 36 metabolites were unambiguously assigned from aqueous extract (**Supplementary Table 3**), including pantothenate, leucine, isoleucine, valine, ethanol, threonine (Thr), lactate, alanine, lysine, acetate, proline, glutamate, succinate, glutamine, glutathione, aspartate, creatine, ethanolamine (EA), choline, O-phosphocholine (PC), sn-glycero-3-phosphocholine (GPC), taurine, glucose, myo-Inositol, glycine, glycerol, ethylene glycol (EG), serine, nicotinamide adenine dinucleotide (NAD^+^), adenine mono/di/tri phosphate (AXP), fumarate, tyrosine, histidine, phenylalanine, formate, and nicotinamide adenine dinucleotide phosphate (NADP^+^). The unsupervised PCA scores plot illustrates the distinct separations of the metabolic profiles among the four groups (data not shown).

### Quantitative Analysis of Characteristic Metabolites

**Figure 4:**
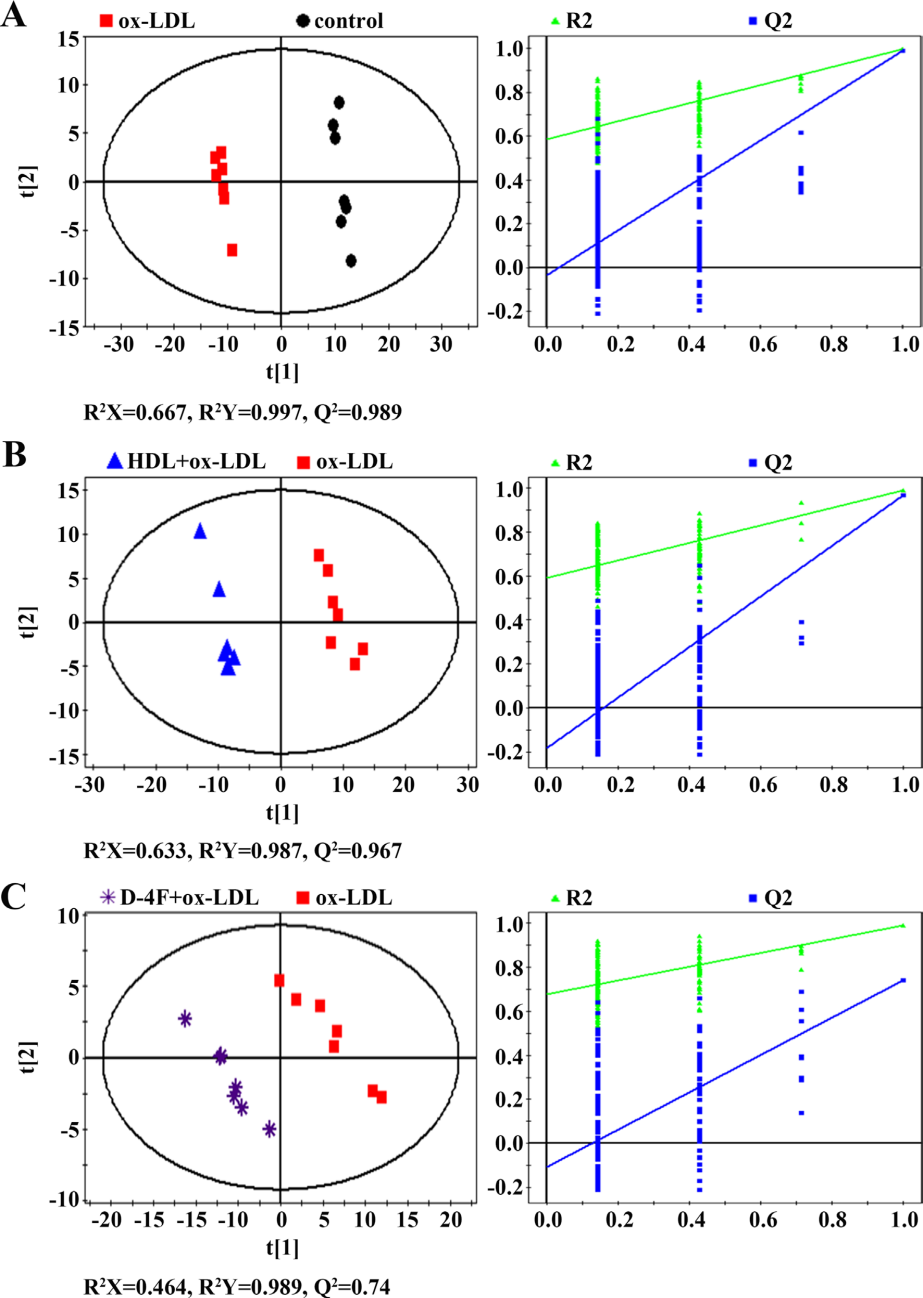
PLS-DA scores plots (left) and cross-validation plots (right) from permutation tests (n = 200) for ^1^H NMR spectra recorded on aqueous extracts derived from endothelial cells. **(A)** ox-LDL vs. control; **(B)** HDL + ox-LDL vs. ox-LDL; **(C)** D-4F + ox-LDL *vs.* ox-LDL.

The supervised PLS-DA was conducted for pair-wise comparisons among the four groups. Distinct segregations of metabolic profiles were observed between control and ox-LDL groups (**Figure 4A**), ox-LDL and HDL + ox-LDL groups (**Figure 4B**), and ox-LDL and D-4F + ox-LDL groups (**Figure 4C**). Both the high values of R^2^Y and Q^2^ and the corresponding cross-validation plots reflect the robustness and credibility of these PLS-DA models, thereby confirming the reliabilities of the metabolic classifications.

To understand the metabolic distinctions between these groups, we quantitatively analyzed the changes in the levels of the assigned metabolites. Relative integrals of these metabolites were calculated from the NMR spectra, and then one-way ANOVA was performed to quantitatively compare the metabolite levels among the four groups. The *p* values were used to represent statistical significances. Meanwhile, VIP scores were computed from the OPLS-DA models. A VIP score > 1 was considered significant for the discrimination of metabolic profiles. Based on the two criteria (*p* < 0.05 and VIP > 1.0), we screened out characteristic metabolites significantly contributing to the metabolic distinctions between these groups.

A total of 15 characteristic metabolites was identified as significantly responsible for the metabolic segregation between control and ox-LDL groups, including succinate, alanine, glutamate, glutamine, aspartate, threonine, glycine, creatine, taurine, choline, PC, GPC, ethanolamine (EA), AXP, and NADP^+^ (**Supplementary Table 4**).

To exploit the effects of HDL and D-4F on the metabolic profiles of endothelial cells impaired by ox-LDL, we identified 16 characteristic metabolites among ox-LDL, HDL + ox-LDL, and D-4F + ox-LDL groups (**Table 1**), including lactate, valine, isoleucine, leucine, alanine, proline, glutamate, glutamine, aspartate, threonine, glycine, creatine, GPC, EA, AXP, and NAD^+^. These characteristic metabolites were mostly involved in three crucial metabolisms: glucose metabolism, amino acid metabolism, and glycerophospholipid metabolism. In addition, several metabolites were labeled as “others,” which were not involved or exclusive in these metabolisms.

**Table 1:** Quantitative analysis of characteristic metabolites among the ox-LDL, HDL + ox-LDL, and D-4F + ox-LDL groups.

Metabolites	Mean ± standard error			One-way ANOVA		VIP scores	
	ox-LDL	HDL + ox-LDL	D-4F + ox-LDL	F	P	VIP1	VIP2
**Glucose metabolism**							
Lactate	0.08 ± 0.01	0.06 ± 0.01**	0.05 ± 0.01***	12.717	2.41e-4	1.46	2.04
**Amino acid metabolism**							
Valine	0.37 ± 0.02	0.34 ± 0.01***	0.38 ± 0.02	13.58	1.64e-4	0.79	0.90
Isoleucine	0.39 ± 0.02	0.36 ± 0.01*	0.40 ± 0.01	11.855	3.58e-4	0.78	0.92
Leucine	0.81 ± 0.07	0.75 ± 0.04*	0.83 ± 0.03	6.424	0.007	0.94	0.92
Alanine	0.93 ± 0.08	0.83 ± 0.05**	0.92 ± 0.07	5.283	0.014	2.01	0.99
Proline	1.01 ± 0.06	0.95 ± 0.02*	1.03 ± 0.05	7.524	0.003	1.38	1.11
Glutamate	3.65 ± 0.10	3.87 ± 0.15**	4.03 ± 0.16***	15.066	8.70e-5	3.54	6.70
Glutamine	2.01 ± 0.08	1.86 ± 0.06**	2.01 ± 0.14	6.319	0.007	2.21	1.08
Aspartate	0.33 ± 0.01	0.34 ± 0.02	0.38 ± 0.04**	8.255	0.002	1.60	2.17
Threonine	0.41 ± 0.03	0.37 ± 0.03*	0.40 ± 0.02	3.891	0.037	2.67	2.37
Glycine	2.34 ± 0.12	2.06 ± 0.09**	2.28 ± 0.20	8.228	0.02	4.92	2.73
Creatine	0.56 ± 0.02	0.62 ± 0.03***	0.59 ± 0.04	9.085	0.001	2.61	2.94
**Glycerophospholipid metabolism**							
GPC	1.66 ± 0.12	2.73 ± 0.17***	1.605 ± 0.100	182.79	5.22e-14	15.65	1.68
EA	0.14 ± 0.01	0.16 ± 0.01***	0.141 ± 0.010	11.794	3.69e-4	3.52	2.01
**Others**							
AXP	0.33 ± 0.02	0.39 ± 0.02***	0.39 ± 0.03***	16.79	4.40e-5	2.61	2.76
NAD^+^	0.07 ± 0.01	0.08 ± 0.01*	0.08 ± 0.01**	7.321	0.004	1.35	1.77

^a^Significant changes of metabolite levels in the HDL + ox-LDL and D-4F + ox-LDL groups compared to the ox-LDL group are assigned: *p < 0.05; **p < 0.01; ***p < 0.001.

^b^Variable importance in the projection (VIP) scores was obtained from the OPLS-DA model. VIP1: HDL + ox-LDL vs. ox-LDL; VIP2: D-4F + ox-LDL vs. ox-LDL.

^c^Characteristic metabolites are highlighted in red or blue for correspondingly representing increased or decreased levels, which were identified on the basis of p < 0.05 from one-way ^e^ANOVA and VIP > 1.0 from the OPLS-DA models.

### Metabolic Alterations in Endothelial Cells Induced by ox-LDL

**Figure 5:**
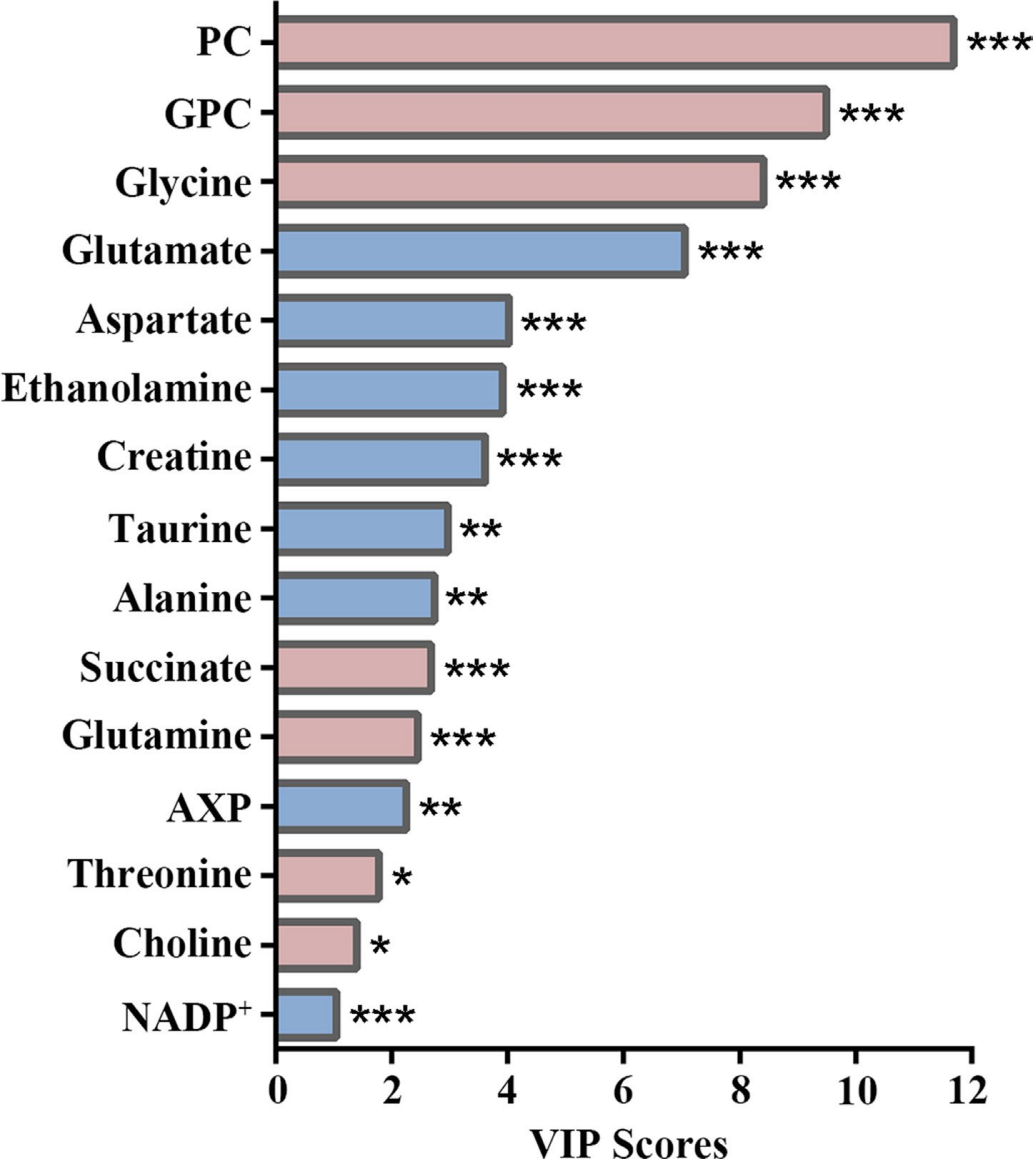
VIP score-rank plot of characteristic metabolites significantly responsible for distinguishing the metabolic profiles between control and ox-LDL groups. Red and blue bars denote the metabolites with increased and decreased levels in the ox-LDL group compared with those in the control group, respectively. Statistical significances: *p< 0.05; **p< 0.01; ***p< 0.001.

The importance of these characteristic metabolites in distinguishing metabolic profiles was ranked by their VIP scores calculated from the OPLS-DA model. As shown in **Figure 5**, PC made the most significant contribution to the metabolic separation between control and ox-LDL groups, followed by GPC and glycine. Additionally, compared with the control group, the ox-LDL group exhibited significantly elevated levels of PC, GPC, glycine, succinate, glutamine, threonine, and choline, and distinctly declined levels of glutamate, aspartate, ethanolamine, creatine, taurine, alanine, AXP, and NADP^+^ (**Supplementary Table 4**).

### Comparison of Metabolic Alterations in Endothelial Cells Induced by HDL and D-4F With the Challenge of ox-LDL

**Figure 6:**
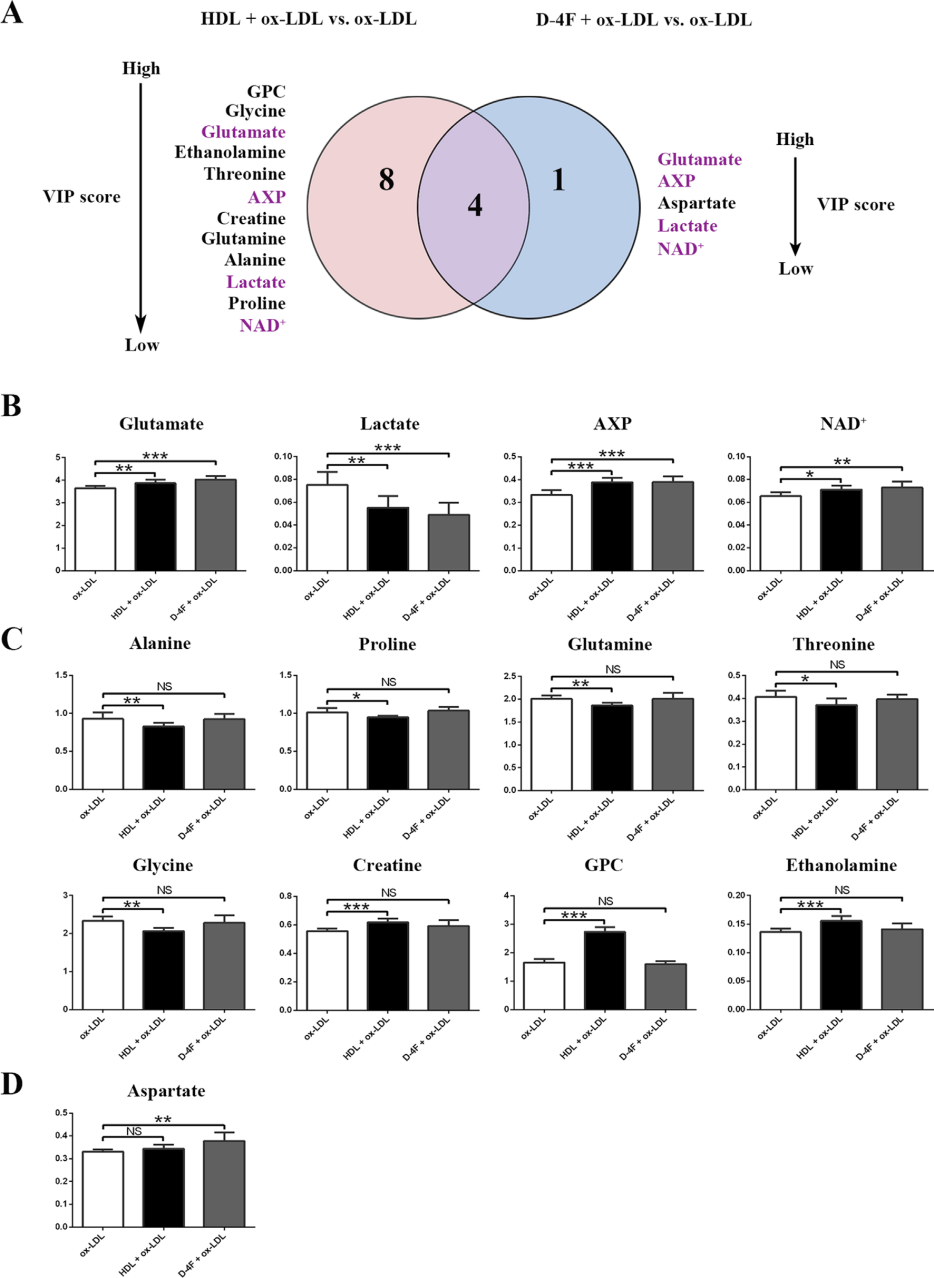
Quantitative analysis of characteristic metabolites significantly responsible for distinguishing the metabolic profiles among ox-LDL, HDL + ox-LDL, and D-4F + ox-LDL groups. **(A)** Venn diagram of characteristic metabolites generated from comparisons of the metabolite levels for HDL + ox-LDL vs. ox-LDL (left-hand circle), and D-4F + ox-LDL vs. ox-LDL (right-hand circle). **(B**–**D)** Histograms of the relative levels of the metabolites in the four groups: **(B)** four common characteristic metabolites shared by both pair-wise comparisons (HDL + ox-LDL vs. ox-LDL; D-4F + ox-LDL vs. ox-LDL); **(C)** eight characteristic metabolites screened specifically for the comparison of HDL + ox-LDL *vs.* ox-LDL; **(D)** one characteristic metabolite screened specifically for the comparison of D-4F + ox-LDL *vs.* ox-LDL. Statistical significances: NS, *p* ≥ 0.05; *p < 0.05; **p < 0.01; ***p < 0.001.

To address the molecular mechanisms of HDL and D-4F underlying the predictive effects of endothelial cells from the perspective of metabolomics, we quantitatively compared the characteristic metabolites identified from the pair-wise analyses of HDL + ox-LDL vs. ox-LDL, and D-4F + ox-LDL vs. ox-LDL basing on the two criterions (*p* < 0.05 and VIP > 1.0). Compared with the ox-LDL group, eight increased metabolites and six decreased metabolites were identified in the HDL + ox-LDL group, whereas four up-regulated metabolites and one down-regulated metabolite were identified in the D-4F + ox-LDL group (**Figure 6A**). Note that four common characteristic metabolites (lactate, glutamate, AXP and NAD^+^) were shared by the two pair-wise comparisons (**Figure 6B**). More significantly, the levels of two metabolites (glutamate and AXP) in HDL + ox-LDL and D-4F + ox-LDL groups displayed obvious recovering tendency toward those in the control group, reflecting the common functions shared by HDL and D-4F. Additionally, the comparison of either HDL + ox-LDL vs. ox-LDL or D-4F + ox-LDL vs. ox-LDL was associated with several specific characteristic metabolites. Aside from the four common characteristic metabolites, the HDL + ox-LDL group displayed the up-regulated levels of creatine and two intermediates in glycerophospholipid metabolism (GPC and ethanolamine), and the down-regulated levels of five amino acids (alanine, proline, glutamine, threonine, and glycine) (**Figure 6C**). Interestingly, only aspartate was specific for the comparison of D-4F + ox-LDL vs. ox-LDL (**Figure 6D**).

Furthermore, these characteristic metabolites were ranked according to their VIP scores (**Figure 6A**). Regarding the metabolic separation of D-4F + ox-LDL vs. ox-LDL, the top three characteristic metabolites were glutamate, AXP, and aspartate, two of which were among the four common characteristic metabolites. Regarding the metabolic separation of HDL + ox-LDL vs. ox-LDL, the top three characteristic metabolites were GPC, glycine, and glutamate. These results indicated that HDL triggered larger-scale metabolic changes in endothelial cells compared with D-4F. Notably, the HDL treatment could change the levels of the intermediates in glycerophospholipid metabolism, whereas the D-4F treatment did not show this capacity.

### Metabolic Pathway Analysis

**Figure 7:**
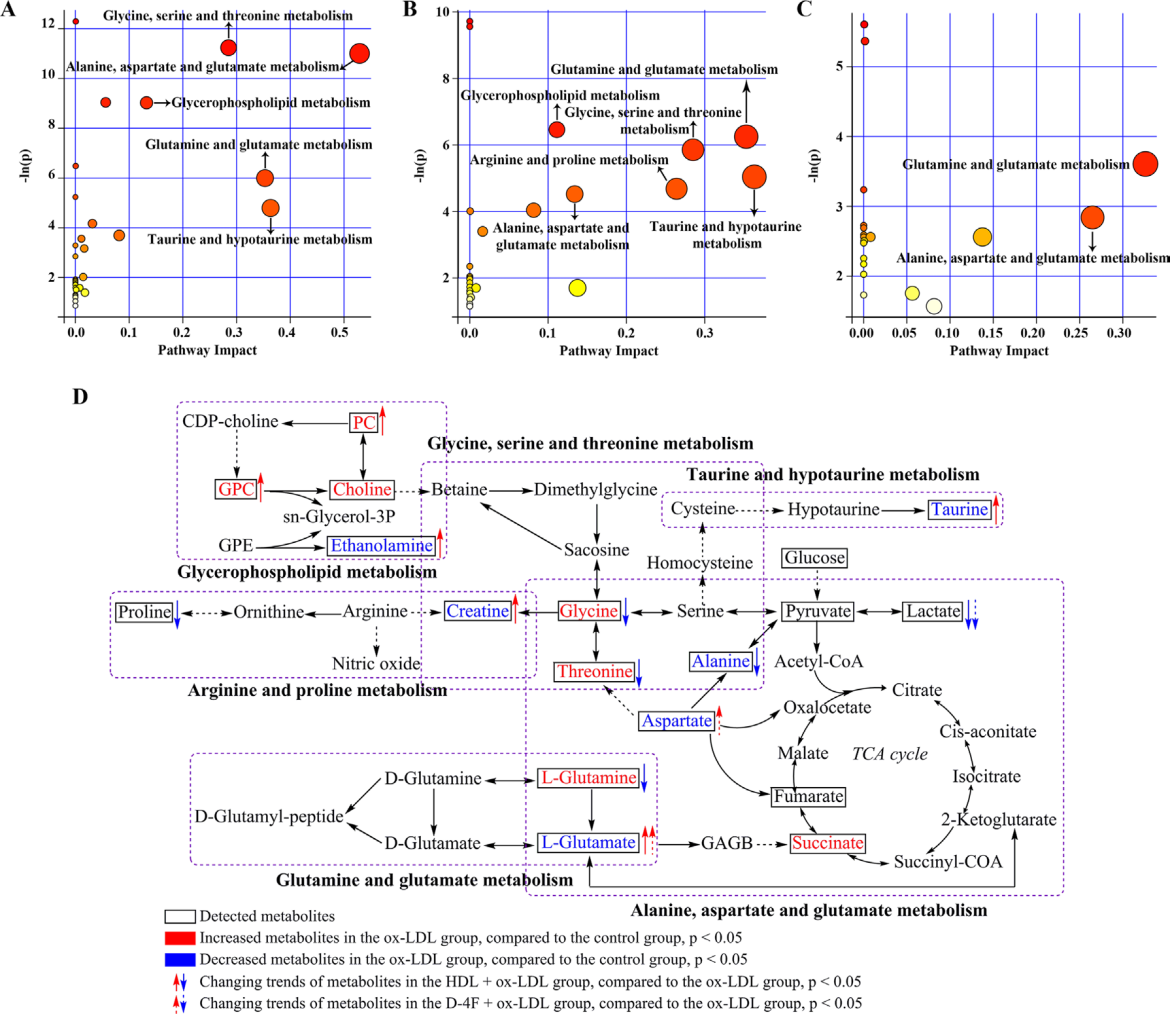
Metabolic pathway analysis based on the characteristic metabolites screened from pair-wise comparisons. Based on both pathway impact values (PIVs) and *p* values, significantly altered metabolic pathways were identified from the pair-wise comparison of **(A)** ox-LDL *vs.* control; **(B)** HDL + ox-LDL *vs.* ox-LDL; **(C)** D-4F + ox-LDL *vs.* ox-LDL. **(D)** Illustration of significantly altered characteristic metabolites induced by either HDL or D-4F against ox-LDL based on the KEG database. The MetaboAnalyst 3.0 web server was used to perform the metabolic pathway analysis. Significantly altered metabolic pathways were identified on the base of the *p* values calculated from the metabolite set enrichment analysis (*p* < 0.001) and the PIVs computed from the topology analysis (PIV > 0.1).

We performed the metabolic pathway analysis by using MetaboAnalyst to identify significant altered metabolic pathways based on these characteristic metabolites. The metabolic pathways were selected on a base of pathway impact value (PIV) >0.1 and probability mass function (*p*) < 0.001. Compared with control, ox-LDL impaired five metabolic pathways, including glycine, serine and threonine metabolism; alanine, aspartate, and glutamate metabolism; glycerophospholipid metabolism; glutamine and glutamate metabolism; and taurine and hypotaurine metabolism (**Figure 7A**).

The metabolic pathway analysis of HDL + ox-LDL *vs.* ox-LDL identified six significantly altered metabolic pathways, including glycerophospholipid metabolism; glutamine and glutamate metabolism; glycine, serine, and threonine metabolism; taurine and hypotaurine metabolism; arginine and proline metabolism; alanine, aspartate, and glutamate metabolism (**Figure 7B**). It is worth mentioning that five of the six metabolic pathways were identical with the ox-LDL-impaired metabolic pathways, excluding arginine and proline metabolism. Interestingly, the metabolic pathway analysis of D-4F+ ox-LDL vs. ox-LDL showed that the D-4F-induced metabolic response was only related to two significantly altered metabolic pathways including glutamine and glutamate metabolism and alanine, aspartate and glutamate metabolism, which might account for the aforementioned observation that D-4F resulted in smaller-scale metabolic changes relative to HDL (**Figure 7C**).

To visualize these metabolic alterations, we projected the identified characteristic metabolites onto a metabolic map based on the Kyoto Encyclopedia of Genes and Genomes (KEGG) database. **Figure 7D** illustrates six significantly altered metabolic pathways, including glycine, serine, and threonine metabolism; taurine and hypotaurine metabolism; glycerophospholipid metabolism; arginine and proline metabolism; glutamine and glutamate metabolism; alanine, aspartate, and glutamate metabolism.

## Discussion

HDL is a complex particle composed of several active components (e.g., apoA-I, PON1 and S1P) exerting protective effects on endothelial cells (Reimers et al., 2011; Levkau, 2015; Soran et al., 2015). However, increasing evidence showed that HDL can lose its protective properties and even develop pro-inflammatory and pro-atherogenic phenotypes in the setting of systemic inflammation (Rosenson et al., 2016). Therefore, HDL is quite difficult to be exploited as exogenous agent substantially used in clinic. As the apoA-I mimetic peptide, 4F could protect apoA-I from oxidative damage *in vitro* and oral D-4F could lower HDL-inflammatory index in high-risk patients *in vivo* (Dunbar et al., 2017; White et al., 2019). Consequently, D-4F is becoming a potential exogenous agent for CVD prevention and treatment owing to the perspective of clinical applications (Navab et al., 2002; Riwanto and Landmesser, 2013). Previous works have demonstrated that D-4F possesses the similar protective effects to HDL on endothelial cells (Riwanto and Landmesser, 2013; Valente et al., 2014; Liu et al., 2017a; Liu et al., 2017b). One question is raised here: What is the difference between the molecular mechanisms underlying the protective effects of D-4F and HDL?

To address this question, we carried out ^1^H NMR-based metabolomic analysis to reveal the molecular mechanisms underlying the protective effects of HDL and D-4F on endothelial cells. Given the crucial role of ox-LDL in endothelial cell dysfunctions (Camont et al., 2013), we assessed the effects of HDL and D-4F for ameliorating ox-LDL-impaired metabolic disorders in endothelial cells. We observed distinctly changed metabolic profiles and markedly altered metabolite levels in endothelial cells, which were caused by the HDL and D-4F treatments. We further screened the characteristic metabolites significantly contributing the metabolic distinctions and identified the profoundly altered metabolic pathways. To the best of our knowledge, this work might represent the mechanistic comparison between the effects of HDL and D-4F against ox-LDL-induced impairment in endothelial cells based on metabolomic profiling.

The ox-LDL treatment significantly disturbed five metabolic pathways, including glycine, serine, and threonine metabolism; alanine, aspartate, and glutamate metabolism; glycerophospholipid metabolism; glutamine and glutamate metabolism; and taurine and hypotaurine metabolism. Similarly, disturbed metabolic pathways were also identified in rabbit aorta in early atherosclerotic plaques (Martin-Lorenzo et al., 2016). Ox-LDL activates NADPH oxidase and triggers intracellular oxidative stress, leading to endothelial cell dysfunctions and atherosclerotic lesion development (Ushio-Fukai, 2006; Madamanchi and Runge, 2013). The present work detected ox-LDL-induced metabolic alterations, further confirming oxidative stress in endothelial cells. First, ox-LDL down-regulated three crucial glucogenic amino acids (alanine, aspartate, and glutamate), which are usually utilized as alternative sources of TCA cycle anaplerosis (Owen et al., 2002). This finding, coupled with the accumulation of succinate, suggests that TCA cycle might be enhanced in response to ox-LDL. Given that ROS are normal byproducts of mitochondrial electronic respiratory chain activity, enhanced tricarboxylic acid cycle (TCA) cycle would enhance ROS production (Lenaz, 2001). Second, glycine, serine, and threonine metabolism is connected with one-carbon unit metabolism, the aberrations of which could decrease EC-SOD expression and result in oxidative stress and CVD susceptibility (Lakshmi et al., 2013). Meanwhile, glycine/one-carbon metabolism also affects intracellular redox balance through folate cycle (Amelio et al., 2014). Third, taurine possesses anti-oxidative capacities (Murakami, 2014). Previous works have demonstrated that taurine supplementation could protect endothelial cells from ox-LDL-induced oxidative stress (Tan et al., 2007; Ulrich-Merzenich et al., 2007). Fourth, aside from the disturbed metabolic pathways related to oxidative stress, we also observed the declined level of NADP^+^ (the sum of NADP^+^ and NADPH) in ox-LDL-treated endothelial cells. The intracellular NADP^+^/NADPH ratio is responsible for maintaining redox status by converting oxidized glutathione to reduced glutathione (World et al., 2006). Taken together, oxidative stress might be a primary event in ox-LDL-triggered abnormal angiogenesis of endothelial cells.

More significantly, the primary metabolic pathways altered by HDL were almost identical to those impaired by ox-LDL. Specifically, five oxidative stress-associated metabolites (glutamate, glutamine, threonine, glycine, and taurine) and creatine exhibited recovering tendencies, illustrating that HDL antagonized ox-LDL-induced oxidative stress. HDL also affected arginine and proline metabolism, which is the main source for producing NO *via* eNOS (Popolo et al., 2014). A previous work has showed the interaction of eNOS with mitochondria could reduce oxidative stress in endothelial cells (Konduri et al., 2015). By contrast, D-4F only affected glutamine and glutamate metabolism, as well as alanine, aspartate, and glutamate metabolism. The protective role of D-4F against ox-LDL-induced oxidative stress was mainly embodied in attenuating the depletion of glutamate and aspartate. Although both HDL and D-4F could alleviate oxidative stress-related metabolic alterations, the protective effects of HDL appear to be greater than those of D-4F.

Angiogenesis, including the proliferation, migration, and capillary formation of endothelial cells, is an energy demanding process (Lapel et al., 2016). ATP production primarily relies on glycolysis in endothelial cells (Eelen et al., 2015). It has been reported that decreased glycolytic ATP production impairs vascular repair and angiogenesis, whereas increased glycolytic ATP production promotes angiogenesis (De Bock et al., 2013). In this study, we observed that ox-LDL inhibited the migration and tube formation of endothelial cells, whereas both HDL and D-4F efficiently alleviated the inhibitory effects of ox-LDL. Furthermore, compared with the control group, the ox-LDL group displayed a reduced AXP level but a relatively stable lactate level. An interpretation of these data is that glycolytic energy production was impaired by ox-LDL. In line with this conjecture, ox-LDL down-regulated GAPDH (a key glycolytic enzyme) expression *via* oxidative stress, resulting in the depletion of cellular energy stores (Sukhanov et al., 2006). Additionally, it was previously indicated that H_2_O_2_-mediated oxidative stress inhibited glucose-dependent ATP synthesis in endothelial cells and caused the decreased endothelial cell viability (Hinshaw and Burger, 1990). We also found that both HDL and D-4F down-regulated lactate levels in endothelial cells and up-regulated AXP and NAD^+^ levels. These results suggest that HDL and D-4F could ameliorate ox-LDL-impaired glycolytic energy production, enhancing the efficiency of utilizing energy to promote angiogenesis. Therefore, we speculate that both HDL and D-4F might play crucial roles in improving the abnormal glycolysis by alleviating oxidative stress in endothelial cells.

Additionally, we also observed some altered intermediates (choline, PC, GPC and enthanolamine) in glycerophospholipid metabolism under the ox-LDL treatment. Alterations in glycerophospholipid metabolism are considered to be a potential metabolic footprint of atherosclerosis progression (Dang et al., 2016). Glycerophospholipid metabolism contains two different routes—one toward phosphatidylcholine and the other toward phosphatidylethanolamine (KEGG map00564) (Dang et al., 2016). Choline metabolites (choline, PC, and GPC) participate in phosphatidylcholine metabolism, which is a precursor of platelet activation factor (PAF) and lysophosphatidylcholines (LPCs). PAF accelerates ROS formation and initiates early atherosclerosis (Brochériou et al., 2000). LPCs also exert important roles in the progression of atherosclerosis (Matsumoto et al., 2007). Accordingly, the promoted phosphatidylcholine route of glycerophospholipid metabolism might be a hallmark of ox-LDL-induced metabolic disorder. However, the reason that the HDL treatment further elevated PC and GPC levels in ox-LDL-treated endothelial cells requires further investigations. On the other hand, ethanolamine is necessary for the biosynthesis of phosphatidylethanolamine (PE), which can be converted to phosphatidylcholine (PC) through three PE methylation steps catalyzed by PE N-methyltransferase (PEMT) (Cole et al., 2011). PEMT deficiency in ApoE^−/−^ mice decreases the PC biosynthesis and attenuates atherosclerosis (Cole et al., 2011). Our work exhibited that the ox-LDL treatment decreased the enthanolamine level, and the HDL treatment attenuated the suppressive effect of ox-LDL on the enthanolamine level. These results illustrate that HDL could ameliorate ox-LDL-disturbed PE route of glycerophospholipid metabolism. Interestingly, D-4F almost did not change the levels of any intermediates in glycerophospholipid metabolism. Previous studies showed that different free fatty acids (FFAs) could be incorporated into newly synthesized PC and PE species, and FFA is an important driving force for PC synthesis in enzymatically modified LDL-loaded primary human skin fibroblasts (Binder et al., 2006). Therefore, we speculated that FFA in both ox-LDL and HDL might promote glycerophospholipid metabolism in endothelial cells. Much differently, D-4F did not affect glycerophospholipid metabolism due to absence of FFA. In the future work, we will further investigate whether D-4F could influence glycerophospholipid metabolism on vascular endothelial cells with presence of FFA in plasma *in vivo*.

Indeed, there are several limitations in the present study. In human plasma, about 90% to 95% of apoA-I are assembled into HDL particles *in vivo* (Rye and Barter, 2004). The purpose of this study was to evaluate whether D-4F could be exploited as a substantially applicable agent for CVD treatment *in vivo*. From the point of application, the mechanistic comparison between D-4F and HDL treatments, rather than D-4F and apoA-I treatments, might provide valuable hints for exploiting the D-4F use in clinic. HDL is an endogenous protective compound against CVD, whereas D-4F is an exogenous agent for CVD prevention and treatment (Dunbar et al., 2017). Previously, we observed that both 100 mg/ml (∼0.3 mmol/L) of HDL and 20 mg/ml (∼8.7 mmol/L) of D-4F treatments displayed similar effects on promoting the proliferation and migration of endothelial cells (Pan et al., 2012; Liu et al., 2017a; Liu et al., 2017b). Given the distinct difference of composition between HDL and D-4F, D-4F shares some but not all of biological effects with HDL, and the protective effects of D-4F are expectedly lower than those of HDL at the same molar concentration. Due to the observably different functional effects of HDL and D-4F at the same weight concentration (e.g., 20 mg/ml) on migration and angiogenesis of endothelial cells (**Figures 1A** and **2A**), it is actually hard to compare the different metabolic changes of endothelial cells and to clarify the mechanistic difference between HDL and D-4F using the same weight concentration. Therefore, we chose the same “functional” concentration of HDL and D-4F in this study, which aimed to address the different effects of D-4F and HDL on cell metabolomics under the circumstance that both had similar effects on migration and angiogenesis of endothelial cells. That is, we tried to compare the metabolomic mechanisms based on the similar effects on endothelial cell protections, rather than using the same molar concentration and weight concentration for the mechanistic comparison between D-4F and HDL treatments. Thus, we used 100 mg/ml of HDL and 20 mg/ml of D-4F to treat endothelial cells in this work. Expectedly, such studies would be of benefit to understanding the mechanistic difference between the protective effects of D-4F and HDL, and further exploiting D-4F as a potential agent for CVD treatment in clinic. However, whether these comparisons were reasonable enough to clarify the differences between HDL and D-4F is still needed to be discussed in depth, because it does not mitigate the possibility that the differences observed and used for mechanistic hypothesis could be explained by the differences in concentrations, not the differences in the action of the compounds. In the future, we will perform a dose-dependent metabolic analysis to compare the effects of D-4F and HDL on cell metabolomics and evaluate both the functional and physical similarities of D-4F to those of HDL. In addition, primary HUVECs were used in this work, which could hardly survive in completely serum-free medium through all the experiments. Thus, endothelial cells were treated with ox-LDL, HDL, or D-4F in 0.5% FBS-ECM in all these groups. The molar concentration of 20 mg/ml of D-4F (∼8.7 mmol/L) was over 500 times higher than that of bovine HDL in 0.5% FBS-ECM (∼ 0.016 mmol/L). Besides, our results also showed that bovine HDL in 0.5% FBS-ECM could not resist ox-LDL-induced impairments in endothelial cells (Liu et al., 2017a; Liu et al., 2017b). Summarily, the influence of bovine HDL on endothelial cells could be ignored. Previous studies showed that apoA-I mimetic peptide 4F could protect apoA-I from oxidative damage (White et al., 2019). Moreover, *in vivo* study also displayed that oral D-4F lowered HDL-inflammatory index in high-risk patients (Dunbar et al., 2017). In this study, we aimed to provide the mechanistic basis for the use of D-4F in clinic. Nevertheless, it is still unknown whether D-4F could also regulate the metabolism of endothelial cells in the presence of HDL *in vivo*. Further works should be conducted to address these issues in the future. Additionally, cholesterol efflux assay is an efficient approach for measuring anti-atherogenic capacities of HDL and D-4F (Rosenson et al., 2013). Expectedly, the cholesterol efflux is closely associated with endothelial cell metabolism. Here, we just compared the metabolomic changes of endothelial cells caused by HDL and D-4F against ox-LDL, and it remains unclear how HDL and D-4F regulate the metabolisms of endothelial cells and which signaling pathways are involved in these regulations. These issues will be addressed in detail in future studies. Furthermore, although both glycolysis and oxidative stress are involved in the angiogenesis of endothelial cells (De Bock et al., 2013; Liu et al., 2017a; Liu et al., 2017b), it is still elusive whether glycerophospholipid metabolism and other metabolic pathways are also involved in angiogenesis of endothelial cells.

In this work, we performed NMR-based metabolomic analysis for aqueous extracts derived from endothelial cells, and screened the characteristic metabolites significantly contributing to the metabolic distinctions between control and ox-LDL groups, also between ox-LDL, HDL + ox-LDL, and D-4F + ox-LDL groups. The results demonstrate that HDL induces larger-scale metabolic changes in endothelial cells compared with D-4F. Basing the screened characteristic metabolites, we identified the primary metabolic pathways significantly altered by the ox-LDL, HDL, and D-4F treatments. Our results reveal that ox-LDL impairs oxidative stress-related metabolic pathways, glycolysis, and glycerophospholipid metabolism, while both HDL and D-4F could alleviate ox-LDL-induced oxidative stress and abnormal glycolysis. HDL could ameliorate more metabolic pathways impaired by ox-LDL compared with D-4F. Specially, HDL could improve ox-LDL-impaired phosphatidylethanolamine route of glycerophospholipid metabolism, whereas D-4F does not possess this capacity.

## Conclusion

In summary, our results demonstrate that NMR-based metabolomic analysis is an efficient approach for comparing the molecular mechanisms underlying the protective effects of HDL and D-4F on endothelial cells. Although D-4F shares the similar effects with HDL on the migration and angiogenesis of endothelial cells, D-4F could not fully cover the molecular mechanisms of HDL. Nevertheless, our work may be of great benefit to further exploiting D-4F to be a clinically applicable agent for CVD treatment.

## Ethics Statement

This study protocol of the isolation of HUVECs was approved by the Ethics Committee of the People’s Liberation Army 174th Hospital. The lipoprotein isolation protocol was approved by the Institutional Review Board of the Affiliated Cardiovascular Hospital of Xiamen University. Healthy volunteers were informed of the study and wrote the consent.

## Author Contributions

WX, MQ, and CH performed the majority of the experiments. PC, WL, QD, SY, and XS supported several experiments and analyzed the data. YG and JZ supervised the research and revised the manuscript. DLiu and DLin designed the experiments and wrote the manuscript. All authors read and approved the final manuscript.

## Funding

This project was supported by grants from the National Natural Science Foundation of China (31200884, 91129713, 81873495) and from the Natural Science Foundation of Fujian (13181444, 2016D016, 2014-ZQN-ZD-2, 2016-ZQN-92).

## Conflict of Interest Statement

The authors declare that the research was conducted in the absence of any commercial or financial relationships that could be construed as a potential conflict of interest.

## Abbreviations

CVD, cardiovascular disease; LDL, low-density lipoprotein; HDL, high-density lipoprotein; apoA-I, apolipoprotein A-I; HUVECs, human umbilical vein endothelial cells; NMR, nuclear magnetic resonance; RCT, reverse cholesterol transport; eNOS, endothelial NO synthase; NO, nitrite oxide; ROS, reactive oxygen species; PCA, principal component analysis; PLS-DA, partial least-squares discriminant analysis; OPLS-DA, orthogonal partial least-squares discriminant analysis; VIP, variable importance in projection.
