# Supplementary material for: Comparison of Mechanisms of Endothelial Cell Protections Between High-Density Lipoprotein and Apolipoprotein A-I Mimetic Peptide
Source: Front Pharmacol. 2019 Jul 19;10:817. doi: 10.3389/fphar.2019.00817 (PMC6659106; doi:10.3389/fphar.2019.00817)
Supplement: Supplementary file 1 [file Table_1.docx]

**Supplementary Table 1.** The value of 100% ± SEM for transwell chamber experiments and scratch-wound healing experiments in Figure 1.

| group comparation | cell migration (% of control)  from the transwell chamber experiments | *p* value | cell migration (% of control) from  the scratch-wound healing experiments | *p* value |
| --- | --- | --- | --- | --- |
| 0 *vs.* 20 μg/ml of HDL | 100.00% ± 1.79 *vs.* 132.98% ± 2.06 | < 0.001 | 100.00% ± 4.49 *vs.* 188.32% ± 2.14 | < 0.001 |
| 0 *vs.* 50 μg/ml of HDL | 100.00% ± 1.79 *vs.* 173.62% ± 2.21 | < 0.001 | 100.00% ± 4.49 *vs.* 212.15% ± 3.51 | < 0.001 |
| 0 *vs.* 100 μg/ml of HDL | 100.00% ±1.79 *vs.* 202.32% ± 2.10 | < 0.001 | 100.00% ± 4.49 *vs.* 242.06% ± 3.94 | < 0.001 |
| 0 *vs.* 5 μg/ml of D-4F | 100.00% ± 1.54 *vs.* 146.53% ± 1.43 | < 0.001 | 100.00% ± 2.75 *vs.* 111.43% ± 3.15 | < 0.001 |
| 0 *vs.* 10 μg/ml of D-4F | 100.00% ± 1.54 *vs.* 172.67% ± 2.49 | < 0.001 | 100.00% ± 2.75 *vs.* 138.93% ± 2.17 | < 0.001 |
| 0 *vs.* 20 μg/ml of D-4F | 100.00% ± 1.54 *vs.* 189.31% ± 0.84 | < 0.001 | 100.00% ± 2.75 *vs.* 171.07% ± 2.92 | < 0.001 |
| control *vs.* ox-LDL | 100.00% ± 1.71 *vs.* 70.28% ± 2.17 | < 0.01 | 100.00% ± 2.74 *vs.* 52.48% ± 2.05 | < 0.001 |
| ox-LDL *vs.* HDL + ox-LDL | 70.28% ± 2.17 *vs.* 129.72% ± 2.57 | < 0.001 | 52.48% ± 2.05 *vs.* 173.29% ± 3.49 | < 0.001 |
| ox-LDL *vs.* D-4F + ox-LDL | 70.28% ± 2.17 *vs.* 117.20% ±1.21 | < 0.001 | 52.48% ± 2.05 *vs.* 155.28% ±3.42 | < 0.001 |
