# Supplementary material for: Comparison of Mechanisms of Endothelial Cell Protections Between High-Density Lipoprotein and Apolipoprotein A-I Mimetic Peptide
Source: Front Pharmacol. 2019 Jul 19;10:817. doi: 10.3389/fphar.2019.00817 (PMC6659106; doi:10.3389/fphar.2019.00817)
Supplement: Supplementary file 2 [file Table_2.docx]

**Supplementary Table 2.** The value of 100% ± SEM for tube formation experiments in Figure 2.

| group comparation | tube formation (% of control) | *p* value |
| --- | --- | --- |
| 0 *vs.* 20 μg/ml of HDL | 100.00% ± 4.43 *vs.* 200.00% ± 2.97 | < 0.01 |
| 0 *vs.* 50 μg/ml of HDL | 100.00% ± 4.43 *vs.* 260.00% ± 3.22 | < 0.001 |
| 0 *vs.* 100 μg/ml of HDL | 100.00% ± 4.43 *vs.* 315.71% ± 3.52 | < 0.001 |
| 0 *vs.* 5 μg/ml of D-4F | 100.00% ± 3.21 *vs.* 135.59% ± 4.27 | < 0.05 |
| 0 *vs.* 10 μg/ml of D-4F | 100.00% ± 3.21 *vs.* 167.80% ± 3.69 | < 0.01 |
| 0 *vs.* 20 μg/ml of D-4F | 100.00% ± 3.21 *vs.* 267.80% ± 5.11 | < 0.001 |
| control *vs.* ox-LDL | 100.00% ± 3.16 *vs.* 39.47% ± 2.63 | < 0.001 |
| ox-LDL *vs.* HDL + ox-LDL | 39.47% ± 2.63 *vs.* 114.04% ± 2.82 | < 0.001 |
| ox-LDL *vs.* D-4F + ox-LDL | 39.47% ± 2.63 *vs.* 91.23% ± 3.08 | < 0.001 |
