# Supplementary material for: Comparison of Mechanisms of Endothelial Cell Protections Between High-Density Lipoprotein and Apolipoprotein A-I Mimetic Peptide
Source: Front Pharmacol. 2019 Jul 19;10:817. doi: 10.3389/fphar.2019.00817 (PMC6659106; doi:10.3389/fphar.2019.00817)
Supplement: Supplementary file 3 [file Table_3.docx]

**Supplementary Table 3.** Indentified metabolites in ^1^H NMR spectra of aqueous extracts from endothelial cells.

| NO. | Metabolites | Moieties | δ ^1^H (ppm) and multiplicy |
| --- | --- | --- | --- |
| 1 | Pantothenate | 0.88(s), 0.92(s) | CH_3_, CH_3_ |
| 2 | Leucine | 0.96(d), 0.97(d), 1.69(m), 1.70(m), 1.73(m), 3.73(m) | α-CH_3_, α-CH_3_, γ-CH, β-CH_2_, α-CH |
| 3 | Isoleucine | 0.94(t), 1.01(d), 1.21(m), 1.42(m), 2.00(m), 3.67(d) | δ-CH_3_, γ-CH_3_, half γ-CH_2_, half γ-CH_2_, β-CH, α-CH |
| 4 | Valine | 0.99(d), 1.05(d), 2.26(m), 3.60(d) | γ-CH_3_, γ-CH_3_, β-CH, α-CH |
| 5 | Ethanol | 1.17 (t), 3.65 (q) | δ-CH_3_, CH_2_ |
| 6 | Thr | 1.30(d), 3.58(d), 4.24(m) | γ-CH_2_, β-CH |
| 7 | Lactate | 1.33(d), 4.11(q) | β-CH_3_, α-CH |
| 8 | Alanine | 1.47(d), 3.78(q) | β-CH_3_, α-CH |
| 9 | Lysine | 1.43(m), 1.49(m), 1.70(m), 1.91(m), 3.02(t), 3.75(t) | half γ-CH_2_, half γ-CH_2_, δ-CH_2_, β-CH_2_, ε-CH_2_, α-CH |
| 10 | Acetate | 1.91(s) | CH_3_ |
| 11 | Proline | 1.99(m) | γ-CH_2_ |
| 12 | Glutamate | 2.08(m), 2.12(m), 2.34(m), 2.37(m), 3.75(m) | half β-CH_2_, half β-CH_2_, half γ-CH_2_, half γ-CH_2_, α-CH |
| 13 | Succinate | 2.42(s) | CH |
| 14 | Glutamine | 2.13(m), 2.45(m), 3.77(t) | γ-CH_2_, β-CH_2_, α-CH |
| 15 | GSH | 2.15(m), 2.55(m), 2.96(m), 3.77(m), 4.56(m) | β-CH_2_, γ-CH_2_, CH_2_-SH, α-CH&CH_2_-NH, CH-NH |
| 16 | Aspartate | 2.69 (dd), 2.81(dd), 3.90(dd) | β-CH_2_, α-CH |
| 17 | Creatine | 3.04(s), 3.93(s) | N-CH_3_, α-CH_2_ |
| 18 | Choline | 3.21(s), 3.51(dd), 4.04(t) | N-(CH_3_)_3_, N-CH_2_, CH_2_OH |
| 19 | PC | 3.22(s), 3.60(t), 4.18(m) | N-(CH_3_)_3_, N-CH_2_, CH_2_OH |
| 20 | GPC | 3.23(s), 3.60(dd), 3.68(dd), 3.87(m), 3.94(m), 4.33(m) | N-(CH_3_)_3_, half ^1^CH_2_, ^2^CH_2_, half ^1^CH_2_, half ^3^CH_2_, half ^3^CH_2_, ^1^CH_2_ |
| 21 | Taurine | 3.27(t), 3.43(t) | ^1^CH_2_, ^2^CH_2_ |
| 22 | Glucose | β(3.24(dd), 3.48(t), 3.90(dd)), α(3.54(dd), 3.71(t), 3.72(dd), 3.83(m)) | β(H_2_, H_3_, H_5_), α(H_2_, H_3_, H_6_) |
| 23 | Myo-Inositol | 3.28(t), 3.53(dd), 3.63(t), 4.07(t) | ^2^CH, ^4,6^CH, ^1,3^CH, ^5^CH |
| 24 | Glycine | 3.57(s) | α-CH_2_ |
| 25 | Glycerol | 3.55(dd), 3.64(dd), 3.77(m) | half ^1^CH_2_, half ^3^CH_2_, ^2^CH |
| 26 | EG | 3.72 (s) | CH_2_ |
| 27 | EA | 3.14 (m), 3.83 (t) | CH_2_NH_2_, CH_2_OH |
| 28 | Serine | 3.83 (dd), 3.96 (m) | CH, CH_2_ |
| 29 | NAD^+^ | 6.03(d), 6.08(s), 8.16(s), 8.20(m), 8.41(s), 8.82(d), 9.13(d), 9.32(s) | NH_2_, NH_2_(CO), δ-CH, β-CH, ^2^CH, γ-CH, α-CH |
| 30 | AXP | 6.14(d), 8.27(s), 8.58(s) | NH2, δ-CH, ^2^CH |
| 31 | Fumarate | 6.51(s) | CH |
| 32 | Tyrosine | 3.05(dd), 3.19(dd), 6.92(d), 7.19(d) | half β-CH_2_, half β-CH^2^, β-CH, α-CH |
| 33 | Phenylalanine | 3.12(dd), 3.30(dd), 3.99(dd), 7.33(d), 7.37(t), 7.43(t) | α-CH, half β-CH_2_, half β-CH_2_, α-CH, β-CH, γ-CH |
| 34 | Histidine | 7.06(s), 7.85(s) | ^5^CH, ^2^CH |
| 35 | Formate | 8.46(s) | CH |
| 36 | NADP^+^ | 6.05 (d), 6.15 (d), 8.16 (s), 8.42 (s), 8.83 (d), 9.11 (d), 9.29 (s) | ^32^CH, ^2^CH, ^12^CH, ^7^CH, ^41^CH, ^43^CH, ^39^CH |

Multiplicity: s, singlet; d, double; t, triplet; q, quartet; m, multiple; dd, double of double.
