# Supplementary material for: Comparison of Mechanisms of Endothelial Cell Protections Between High-Density Lipoprotein and Apolipoprotein A-I Mimetic Peptide
Source: Front Pharmacol. 2019 Jul 19;10:817. doi: 10.3389/fphar.2019.00817 (PMC6659106; doi:10.3389/fphar.2019.00817)
Supplement: Supplementary file 4 [file Table_4.docx]

**Supplementary Table 4.** Quantitative analysis of differential metabolites between control and ox-LDL groups.

| **Metabolites** | **Mean ± Standard error** | |  | **t-test** |
| --- | --- | --- | --- | --- |
|  | **control** | **ox-LDL** |  | **P** |
| **Glucose metabolism** | | |  |  |
| Succinate | 0.22 ± 0.01 | 0.28 ± 0.01 |  | 2.23e-8 |
| **Amino acid metabolism** | |  |  |  |
| Alanine | 1.03 ± 0.08 | 0.93 ± 0.08 |  | 0.024 |
| Glutamate | 4.42 ± 0.33 | 3.65 ± 0.10 |  | 1.81e-4 |
| Glutamine | 1.79 ± 0.08 | 2.01 ± 0.08 |  | 5.60e-5 |
| Aspartate | 0.51 ± 0.04 | 0.33 ± 0.01 |  | 3.42e-9 |
| Threonine | 0.38 ± 0.02 | 0.41 ± 0.03 |  | 0.017 |
| Glycine | 1.81 ± 0.13 | 2.34 ± 0.12 |  | 6.94e-7 |
| Creatine | 0.65 ± 0.05 | 0.56 ± 0.02 |  | 0.001 |
| Taurine | 0.90 ± 0.06 | 0.80 ± 0.06 |  | 0.005 |
| **Glycerophospholipid metabolism** | | |  |  |
| PC | 3.55 ± 0.32 | 4.86 ± 0.39 |  | 4.00e-6 |
| GPC | 0.95 ± 0.10 | 1.66 ± 0.12 |  | 4.48e-9 |
| EA | 0.19 ± 0.01 | 0.14 ± 0.01 |  | 2.15e-7 |
| **Others** |  |  |  |  |
| AXP | 0.38 ± 0.04 | 0.33 ± 0.02 |  | 0.007 |
| NADP^+^ | 0.04 ± 0.01 | 0.03 ± 0.01 |  | 3.14e-4 |
| Glutathione | 0.49 ± 0.02 | 0.43 ± 0.05 |  | 0.01 |
